# Clinical Outcomes of Platelet-Rich Plasma in Otology: A Systematic Review of Tympanoplasty, Myringoplasty, and Hearing Loss Management

**DOI:** 10.7759/cureus.104045

**Published:** 2026-02-22

**Authors:** Sara Bayounos, Rahaf G Altwairqi, Abdulrahman F Kabli, Mazen A Hamed, Sarah S Almohammdi, Reema Althubaiti, Bayan B Altowairqi

**Affiliations:** 1 Otolaryngology - Head and Neck Surgery, King Abdullah Medical City, Makkah, SAU; 2 Otolaryngology - Head and Neck Surgery, Alhada Armed Forces Hospital, Taif, SAU; 3 Otolaryngology - Head and Neck Surgery, Makkah Health Cluster, Makkah, SAU; 4 Medicine and Surgery, Umm Al-Qura University, Makkah, SAU; 5 College of Medicine, Taif University, Taif, SAU

**Keywords:** myringoplasty, otologic surgery, platelet-rich plasma, sensorineural hearing loss, tympanoplasty

## Abstract

Platelet-rich plasma (PRP), an autologous concentrate of platelets and growth factors, has been explored as an adjunct in otologic procedures, including tympanoplasty, myringoplasty, and treatment of sudden sensorineural hearing loss (SNHL). This systematic review evaluated reported clinical outcomes associated with PRP in these settings. A literature search was conducted in March 2025 across PubMed, Scopus, Web of Science, EMBASE, and the Cochrane Library. Eligible studies were English-language human studies (2015-2025) with original clinical outcome data from randomized or comparative observational designs. Due to methodological heterogeneity, findings were synthesized narratively. Six studies (n = 349 patients) were included: five addressing tympanic membrane repair and one addressing idiopathic sudden SNHL. In myringoplasty/tympanoplasty-related studies, PRP was generally associated with higher graft uptake and/or faster healing than comparator approaches; where reported, closure outcomes included 85.7% versus 60% in one comparative study, and several studies reported between-group differences with p < 0.05. Reported postoperative complications were low, and no major PRP-related safety concerns were identified in the included studies. In SNHL, one comparative study suggested hearing improvement with intratympanic PRP that was comparable to, or greater than, steroid therapy, but evidence remains limited to short-term follow-up and a small number of studies. Current evidence supports PRP as a feasible and apparently safe adjunct in otologic practice, particularly for tympanic membrane repair. However, certainty regarding the magnitude of benefit and long-term auditory outcomes remains limited by small sample sizes, heterogeneity in PRP preparation/application, and variability in study design. Standardized protocols and larger, well-designed randomized trials are needed.

## Introduction and background

Otologic surgeries such as tympanoplasty and myringoplasty are commonly performed to repair tympanic membrane perforations and manage chronic otitis media. Despite technical advances, clinically relevant challenges remain, particularly incomplete graft uptake, delayed epithelialization, and variable postoperative hearing outcomes. In this context, platelet-rich plasma (PRP) - an autologous platelet concentrate containing growth factors such as Platelet-Derived Growth Factor (PDGF), Vascular Endothelial Growth Factor (VEGF), and Transforming Growth Factor-Beta (TGF-β) - has been proposed as a potential adjunct to support tissue repair and reduce postoperative morbidity [1, 2].

The biological rationale for platelet-rich plasma in otology is plausible but should be interpreted within the specific constraints of ear surgery. Unlike highly vascularized tissues, where it has been widely studied, the tympanic membrane and middle ear represent a distinct microenvironment characterized by thin-layered architecture, limited local vascularity in some regions, and sensitivity to infection, moisture, and ventilation dynamics. These factors may influence how platelet-rich plasma-derived cytokines and growth factors affect epithelial migration, fibroblast activity, graft integration, and functional recovery. Therefore, assumptions of direct translatability from other surgical specialties to otologic tissues require condition-specific evaluation [1, 2].

Available otologic studies suggest a possible benefit, particularly in tympanic membrane repair. Prior reports have described higher closure rates, faster healing, and reduced postoperative complications when PRP is added to myringoplasty/tympanoplasty techniques [2-6]. Platelet-rich plasma has also been explored for inner ear disorders such as sensorineural hearing loss (SNHL), with preliminary findings suggesting potential auditory improvement in selected patients after intratympanic administration [1]. However, this evidence remains early and heterogeneous, with differences in patient selection, comparator arms, and follow-up periods.

Several methodological issues limit definitive interpretation of the current literature. Across studies, platelet-rich plasma preparation and application vary substantially (e.g., centrifugation protocols, platelet concentration, activation methods, topical vs injectable delivery), and many studies are small, single-center, and non-randomized [3-5]. In addition, outcome definitions are not uniform, with variability in how graft uptake, hearing gain, and complications are measured and reported. These limitations create uncertainty regarding effect size, reproducibility, and generalizability in routine otologic practice [6-9].

Accordingly, the key research gap is not only whether it may be beneficial, but under what clinical and technical conditions it provides a meaningful advantage over standard care. A structured synthesis focused specifically on otologic outcomes is therefore needed to evaluate evidence quality, identify consistent signals of benefit, and clarify unresolved questions related to protocol standardization and comparative effectiveness [1, 2, 7].

This systematic review aims to evaluate the clinical outcomes of platelet-rich plasma in tympanoplasty, myringoplasty, and hearing loss management, with emphasis on graft uptake, healing, auditory outcomes, safety, and sources of heterogeneity. By consolidating available evidence, this review seeks to support evidence-informed otologic decision-making and define priorities for future high-quality trials [1, 2, 7].

## Review

Methodology

Review Design and Objectives

This systematic review evaluated clinical outcomes associated with platelet-rich plasma use in otologic interventions, specifically tympanoplasty, myringoplasty, and intratympanic treatment for SNHL. The review was conducted and reported in accordance with the PRISMA 2020 statement [10]. The primary objective was to determine whether PRP use was associated with differences in graft uptake or closure, postoperative healing, audiological improvement, and complication profiles compared with non-PRP approaches.

Protocol and Registration

A protocol was developed before formal screening and data extraction, including the research question, eligibility criteria, outcomes of interest, databases, and appraisal tools. However, the protocol was not prospectively registered in PROSPERO or an equivalent platform. This is acknowledged as a methodological limitation because registration improves transparency and reproducibility. To reduce selective reporting risk despite non-registration, eligibility criteria and primary outcomes were defined a priori and were not substantively altered after screening commenced. Future updates of this review will be prospectively registered.

Eligibility Criteria

Eligibility criteria were defined using the PICOS framework. Included studies enrolled human participants undergoing otologic interventions in which PRP was applied intraoperatively, topically, or via intratympanic injection, with comparison to conventional treatment without platelet-rich plasma or alternative adjunctive approaches. Eligible studies were required to report at least one clinical outcome relevant to graft uptake or closure, hearing improvement, healing trajectory, complications, or patient-reported outcomes. We included original peer-reviewed studies published in English between January 2015 and March 2025, including randomized controlled trials and comparative observational designs (prospective or retrospective cohorts and case-control studies). Animal studies, in vitro studies, conference abstracts without full data, case reports, reviews, editorials, and studies without extractable PRP-specific otologic outcomes were excluded.

Search Strategy

A comprehensive search was performed in March 2025 across PubMed/MEDLINE, Scopus, Web of Science, EMBASE, and the Cochrane Library. Search terms combined controlled vocabulary and free-text keywords related to PRP and otologic surgery, including “platelet-rich plasma,” “PRP,” “tympanoplasty,” “myringoplasty,” “sensorineural hearing loss,” “hearing restoration,” and “middle ear surgery,” linked using Boolean operators (AND/OR). Search syntax was adapted to database indexing structures, with MeSH-oriented construction in PubMed and free-text expansions in Scopus and Web of Science. Reference lists of relevant reviews and included studies were also screened manually to identify additional eligible publications.

Study Selection

Search records were imported into EndNote X9 (Clarivate, London, UK), and duplicates were removed through automated and manual verification. Study selection proceeded in two stages: initial title/abstract screening followed by full-text eligibility assessment. Two reviewers independently performed both stages. Disagreements were resolved through discussion and, when needed, third-reviewer adjudication. Inter-reviewer agreement was quantified using Cohen’s kappa (κ) at the title/abstract and full-text stages to support screening reliability. The final selection process was documented using a PRISMA 2020 flow diagram [10].

Data Extraction

Data extraction was completed independently by two reviewers using a standardized Microsoft Excel template. Extracted variables included first author, publication year, country, study design, sample size, participant characteristics, PRP preparation and application method, surgical or interventional details, comparator characteristics, outcome definitions, follow-up duration, and statistical findings. Clinical outcomes included graft closure or uptake, hearing metrics (such as pure-tone measures and air-bone parameters where reported), postoperative complications, and recovery indicators. Extraction discrepancies were resolved by consensus, with third-reviewer input when required.

Quality Assessment

**Figure 1:**
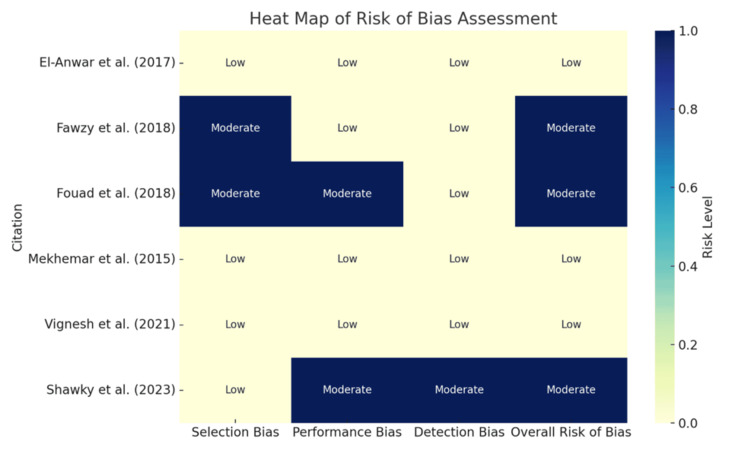
Heat Map of Risk of Bias Assessment

Risk of bias was assessed independently by two reviewers using design-appropriate validated tools. Randomized controlled trials were evaluated using the Cochrane Risk of Bias tool (RoB 2.0) [11], and observational studies were appraised using the Newcastle-Ottawa Scale (NOS). Domain-level judgments were synthesized into an overall bias interpretation to classify studies as lower or higher concern in internal validity. Disagreements in quality ratings were resolved by consensus. Risk-of-bias findings were incorporated into the interpretation of results, such that findings from studies with moderate concerns in selection, performance, or detection domains were interpreted more cautiously when considering effect magnitude and certainty (Figure 1).

Data Synthesis

A formal meta-analysis was not performed because heterogeneity was substantial across clinical context, methodology, and outcome reporting. Clinical heterogeneity arose from differences in indication (tympanic membrane repair versus SNHL), intervention delivery, and comparator strategy. Methodological heterogeneity reflected the mixture of randomized and non-randomized designs. Reporting heterogeneity included non-uniform outcome definitions, variable follow-up intervals, and inconsistent statistical details across studies.

Given these constraints, evidence was synthesized narratively with thematic grouping by procedure type. To improve analytical depth beyond descriptive summary, a semi-quantitative approach was applied by summarizing the direction of effect across key outcomes and reporting available comparative estimates and p-values where provided, without calculating pooled effect sizes. No de novo effect measures (e.g., risk ratios with 95% confidence intervals) were reconstructed from published data, because several studies lacked sufficiently complete and harmonized raw outcome data for valid cross-study calculation. Accordingly, results are presented as study-level reported findings and directional patterns, with explicit distinction between statistically supported comparisons and outcomes reported without inferential statistics.

Ethical Considerations

This review relied exclusively on secondary analysis of published studies and involved no direct patient participation or primary data collection; therefore, institutional ethical approval was not required. The review process was conducted according to accepted academic standards for evidence synthesis.

Results

Study Selection and Overall Yield

**Figure 2:**
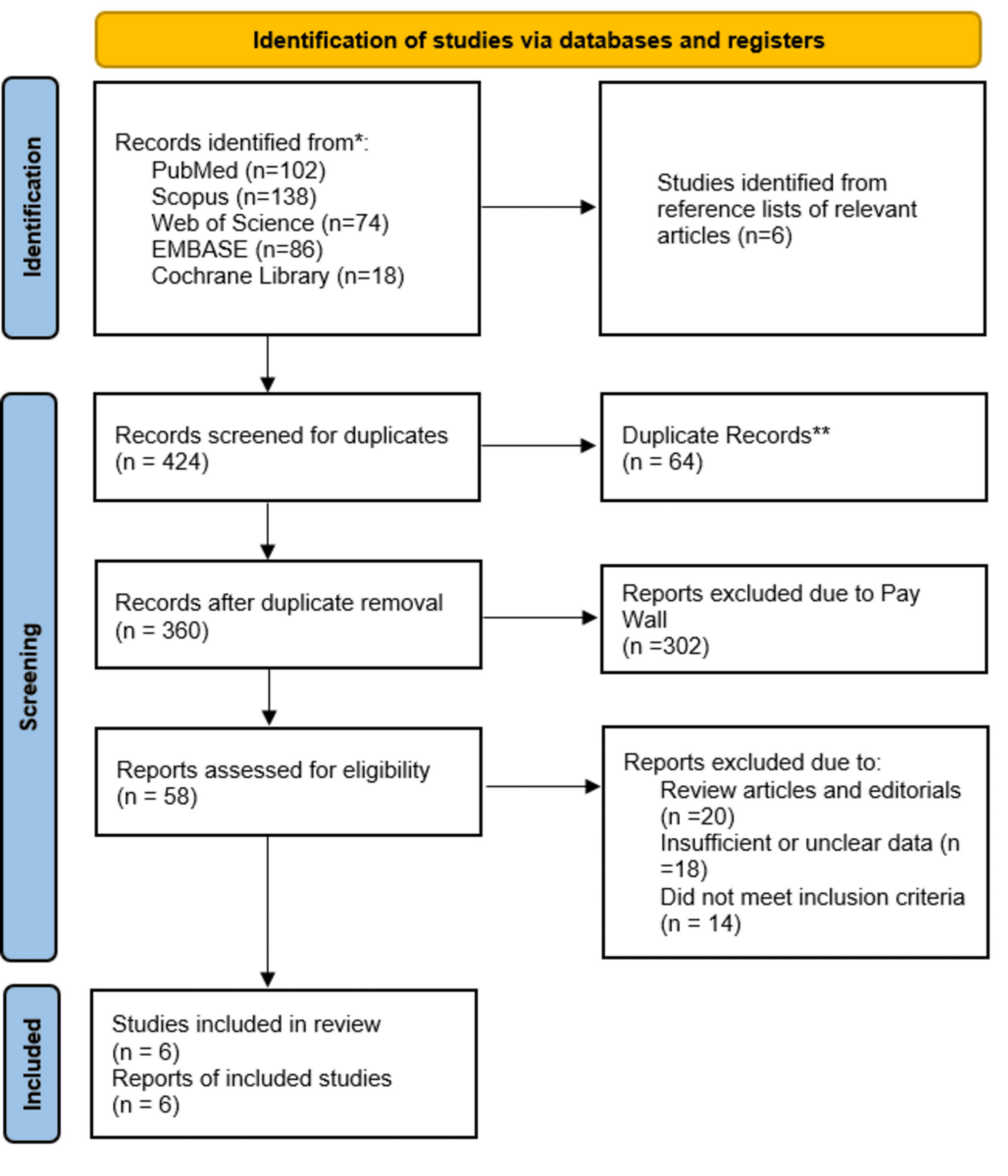
PRISMA flow chart for the search process

The review identified records from PubMed (n=102), Scopus (n=138), Web of Science (n=74), EMBASE (n=86), and Cochrane Library (n=18), with additional records identified through reference-list screening (n=6), yielding 424 records before duplicate removal. After removing duplicates (n=64), 360 records remained for screening. Following title/abstract screening and full-text assessment, six studies met the inclusion criteria. Full-text exclusions were mainly due to review/editorial format (n=20), insufficient or unclear outcome data (n=18), and failure to meet inclusion criteria (n=14) (Figure 2).

Characteristics of Included Studies

Six studies published between 2017 and 2023 were included (total n=349): one randomized controlled trial, four prospective non-randomized studies, and one retrospective comparative study [3,9,12-15]. Five studies addressed tympanic membrane repair contexts (predominantly myringoplasty/fat-graft myringoplasty), and one addressed intratympanic platelet-rich plasma for idiopathic sudden Sensorineural Hearing Loss [15]. Sample sizes ranged from 30 to 100, and assessed outcomes included graft uptake/closure, healing/complications, and hearing-related endpoints (Table 1).

**Table 1:** Characteristics of the Included Studies (n=6) PRP: Platelet-rich plasma; CSOM: Chronic suppurative otitis media; ISSNHL: Idiopathic sudden sensorineural hearing loss; TM: Tympanic membrane; HA: Hyaluronic acid.

Authors	Year	Aim	Sample	Design	Key Findings
El-Anwar et al. [12]	2017	To evaluate PRP as an office-based technique for repairing small tympanic membrane perforations.	30 patients with small TM perforations	Prospective clinical study	High success rate of graft closure with minimal complications; PRP feasible as an outpatient repair method.
Fawzy et al. [13]	2018	To assess the efficacy of PRP added to fat grafts in myringoplasty for CSOM.	60 patients with chronic suppurative otitis media	Prospective comparative study	Significantly improved graft uptake rates when PRP was combined with fat graft.
Fouad et al. [14]	2018	To compare PRP and hyaluronic acid as adjuncts in fat graft myringoplasty.	69 patients divided into three groups (PRP, HA, control)	Retrospective comparative study	PRP and HA both improved closure rates over control; PRP showed slightly higher success.
Mekhemar et al. [3]	2020	To evaluate the topical use of autologous PRP in enhancing myringoplasty outcomes.	40 patients undergoing myringoplasty	Prospective clinical study	Improved healing and reduced infection rates in PRP group.
Vignesh et al. [9]	2021	To determine the effect of PRP on graft uptake in myringoplasty.	50 patients (25 PRP, 25 control)	Single-blinded randomized controlled trial	Higher graft success rate in the PRP group compared to the control group.
Shawky [15]	2023	To compare intratympanic PRP and steroids in treating sudden sensorineural hearing loss.	100 patients with ISSNHL	Prospective comparative clinical study	PRP showed comparable or better hearing improvement than steroids; well tolerated.

Quantitative Outcome Signals

For graft-related outcomes, comparative studies generally showed a directional effect favoring PRP, with statistical significance reported in three comparisons. Fawzy et al. [13] reported higher graft uptake with PRP plus fat graft (p<0.05). Fouad et al. [14] reported closure rates of 85.7% in PRP versus 60% in controls (absolute difference 25.7 percentage points; p<0.05). Vignesh et al. [9] reported higher graft success in the PRP arm (p<0.05). El-Anwar et al. [12] and Mekhemar et al. [3] reported favorable healing/complication trends, but inferential statistics were not fully reported.

For hearing outcomes, reporting was less complete. In tympanic membrane repair cohorts, hearing improvement was described in PRP-treated groups [3,12], but standardized effect estimates were inconsistently reported. In SNHL, Shawky [15] reported better or comparable hearing gain with PRP versus steroids; however, detailed effect-size and p-value reporting was incomplete (Table 2).

**Table 2:** Summary of the Clinical Outcomes ISSNHL: Idiopathic sudden sensorineural hearing loss

Authors	Procedure Type	Outcome Measures	PRP Group Result	Control Group Result	p-value / Statistical Significance
El-Anwar et al. [12]	Myringoplasty	Graft closure rate, complications	High closure rate, minimal complications	Not applicable	Not reported
Fawzy et al. [13]	Myringoplasty	Graft uptake rate	Significantly higher uptake rate	Lower uptake rate	p < 0.05
Fouad et al. [14]	Fat graft myringoplasty	Closure rate	85.7%	60%	p < 0.05
Mekhemar et al. [3]	Myringoplasty	Healing rate, infection	Improved healing, fewer infections	Lower healing rate, more infections	Not reported
Vignesh et al. [9]	Myringoplasty	Graft success rate	Higher success rate	Lower success rate	p < 0.05
Shawky [15]	ISSNHL Treatment	Hearing improvement	Better hearing gain	Steroids: moderate gain	Not reported

Risk of Bias and Its Impact on Certainty

Risk-of-bias assessment classified three studies as low overall risk (El-Anwar et al. [12], Mekhemar et al. [3], Vignesh et al. [9]) and three as moderate overall risk (Fawzy et al. [13], Fouad et al. [14], Shawky [15]) (Table 3). Moderate concerns were concentrated in selection and/or performance domains, especially in non-randomized and retrospective designs.

**Table 3:** Risk of Bias and Quality Assessment

Authors	Study Design	Selection Bias	Performance Bias	Detection Bias	Overall Risk of Bias
El-Anwar et al. [12]	“Prospective clinical study”	L^*^	L	L	L
Fawzy et al. [13]	“Prospective comparative study”	M^*^	L	L	M
Fouad et al. [14]	“Retrospective comparative study”	M	M	L	M
Mekhemar et al. [3]	“Prospective clinical study”	L	L	L	L
Vignesh et al. [9]	“Randomized controlled trial”	L	L	L	L
Shawky [15]	“Prospective comparative study”	L	M	M	M
L: Low, M: Moderate					

Integrated Synthesis of Findings

Across tympanic membrane repair studies, PRP showed a recurrent favorable direction for graft uptake/closure and healing outcomes, with statistically significant between-group differences reported in several comparisons [9,13,14]. However, effect-size precision was limited by heterogeneity in design, outcome definitions, and incomplete statistical reporting in some studies [3,12].

For SNHL, evidence is currently limited to one comparative study [15]. Overall, the evidence pattern supports a favorable directional signal for PRP in membrane-repair settings, while statistical robustness remains variable across studies.

Discussion

This review identified a recurrent directional signal favoring PRP in otologic practice, particularly for tympanic membrane repair, but the strength of inference remains moderate rather than definitive. Across included studies, PRP-treated groups generally showed higher graft uptake/closure and faster healing trends, and several comparative analyses reported statistical significance [16-18]. However, the evidence base is small, methodologically heterogeneous, and dominated by non-randomized designs, so effect magnitude and reproducibility should be interpreted cautiously.

The biological rationale for platelet-rich plasma in otology is plausible but still incompletely characterized in clinical studies. PRP contains platelet-derived mediators (e.g., PDGF, TGF-β, VEGF) that may support angiogenesis, fibroblast activity, epithelial migration, and tissue remodeling, all relevant to tympanic membrane healing [19,20]. At the same time, biochemical plausibility alone does not confirm clinical causality. In practice, observed benefit may result from a composite of mechanisms, including growth-factor signaling, fibrin-matrix scaffold effects, local moisture/seal optimization, and improved graft apposition during surgery. Current studies rarely disentangle these contributions, limiting mechanistic certainty.

An important nuance is that PRP is not a single standardized intervention. Studies differ in centrifugation method, platelet concentration, leukocyte content, activation strategy, delivery route (topical vs injectable), and timing of administration. These variables may materially influence bioactivity, growth-factor kinetics, inflammatory profile, and, therefore, clinical response. For example, leukocyte-rich versus leukocyte-poor preparations could plausibly produce different inflammatory and reparative trajectories, yet most otologic studies do not report sufficient preparation details for dose-response or subtype analysis. This methodological variability is likely one reason why cross-study synthesis remains interpretively constrained despite broadly favorable trends.

The comparative signal is strongest for tympanic membrane repair, where several studies reported better closure or graft success in PRP groups [21]. Even here, caution is needed because outcome definitions and follow-up windows are inconsistent, and exact effect estimates are not uniformly reported. The single randomized study strengthens confidence in a potential benefit [9], but most supporting data are observational; therefore, conclusions should emphasize the likelihood of benefit rather than proof of superiority.

For SNHL, the evidence remains preliminary. The included comparative study suggests intratympanic platelet-rich plasma may be comparable or possibly favorable versus steroids in short-term hearing recovery [15], but this question is far from settled. SNHL is etiologically heterogeneous, and response may depend on treatment timing, baseline severity, and outcome metric selection. Additional randomized, adequately powered studies with standardized audiologic endpoints are required before PRP can be positioned beyond investigational use in this setting.

Alternative explanations for positive findings should also be considered. Apparent improvements could be influenced by surgeon experience and operative technique, perioperative care differences, selection of less complex perforations for PRP arms, regression to the mean in hearing outcomes, or incomplete blinding in outcome assessment [22]. In non-randomized and retrospective designs, these factors can inflate apparent treatment effects. Our bias appraisal supports this caution, as moderate concerns were present in several studies, especially in selection and performance domains.

From a safety perspective, short-term tolerability appears acceptable across included reports, with no major PRP-related adverse events consistently identified. Nonetheless, the absence of observed short-term harm is not equivalent to established long-term safety or effectiveness. Follow-up duration was limited in many studies, and adverse-event reporting standards were not fully uniform.

Taken together, platelet-rich plasma should be interpreted as a potentially useful but still evolving adjunct in otologic surgery, with the most credible current signal in tympanic membrane repair. At present, the evidence supports feasibility and short-term clinical promise rather than conclusive long-term efficacy. Future work should prioritize protocol harmonization (including explicit PRP composition/reporting standards), core outcome sets, stratified analyses by perforation characteristics and patient profile, and multicenter randomized trials powered for both anatomical and functional endpoints.

Limitations

This review has several important limitations that constrain interpretive certainty. First, although PRISMA 2020 procedures were followed [10], the protocol was developed a priori but not prospectively registered (e.g., PROSPERO), which limits external verification of procedural decisions. We mitigated this by predefining eligibility and outcomes and avoiding substantive post hoc changes, but non-registration remains a methodological weakness.

Second, comprehensiveness was affected by full-text accessibility constraints. A substantial number of potentially relevant records could not be fully assessed because articles were closed-access/paywalled at the time of screening. This may have introduced availability bias and reduced the completeness of the evidence base. Accordingly, findings should be interpreted as a synthesis of accessible evidence rather than an exhaustive capture of all eligible global studies.

Third, the included evidence base was small and heterogeneous, with only one randomized trial and several non-randomized/retrospective studies. Study-level variability in PRP preparation (centrifugation protocols, platelet/leukocyte composition, activation methods), intervention delivery, comparators, and follow-up intervals limited cross-study comparability and precluded valid pooled meta-analysis. Even with semi-quantitative synthesis (direction-of-effect reporting), certainty in effect magnitude remains limited.

Fourth, statistical reporting was incomplete in several studies, including inconsistent provision of exact effect estimates and p-values. This restricts robust comparative inference and increases the risk that directional impressions appear stronger than the underlying statistical support.

Fifth, publication bias is a meaningful concern, particularly in a field characterized by small, single-center studies and a predominance of positive findings. Negative or null studies may be underreported, potentially inflating perceived benefit. Language restriction to English-only publications may also have introduced language bias and excluded relevant evidence from other regions.

Finally, long-term outcomes were insufficiently represented. Most included studies emphasized short-term graft or early hearing outcomes, limiting conclusions regarding durability, late complications, and sustained auditory benefit. For these reasons, the present conclusions should be read as cautious and provisional, pending larger, prospectively registered, methodologically standardized randomized studies.

## Conclusions

Current evidence suggests possible, rather than definitive, benefits of PRP as an adjunct in otologic care. Based on six included studies, the most supported signal is in myringoplasty/fat-graft tympanic membrane repair, where PRP was associated with improved short-term graft closure/uptake trends and low reported short-term complication rates in several comparisons. Evidence for hearing improvement is less certain because reporting was heterogeneous and, in SNHL, conclusions are currently based on limited comparative data. Therefore, long-term auditory restoration and cochlear-level regenerative effects remain investigational. Importantly, this review should not be interpreted as proving efficacy across all otologic procedures. The included evidence base is small, methodologically heterogeneous, and predominantly non-randomized. In addition, no included study directly evaluated formal tympanoplasty as a distinct analytical stratum in a way that supports strong procedure-specific inference. Accordingly, PRP is best viewed at present as a potentially useful but still evolving adjunct, not a standard-of-care replacement.

Future research should prioritize prospectively registered, multicenter randomized trials with standardized platelet-rich plasma protocols (including platelet/leukocyte characterization, activation method, and delivery route), predefined core outcome sets, and longer follow-up. Quantification of PRP biologic composition (including growth-factor profiling where feasible) and transparent reporting of effect sizes will be essential to define which patients and procedures derive clinically meaningful benefit.

## Appendices

Appendix A. Full search strategies and retrieval details

A1. Search Scope and Date

A comprehensive literature search was conducted in March 2025 across PubMed/MEDLINE, Scopus, Web of Science Core Collection, EMBASE, and the Cochrane Library. Backward citation screening of included studies and relevant reviews was also performed to identify additional eligible records.

A2. Search Concepts

The search combined terms related to: (1) platelet-rich plasma (PRP), (2) otologic surgery and tympanic membrane repair, and (3) hearing loss, including sudden sensorineural hearing loss (SSNHL/ISSNHL).

Controlled vocabulary (where available) and free-text keywords were used with Boolean operators.

A3. Database-Specific Strategies and Retrieval Counts

A3.1 PubMed/MEDLINE

Date searched: 31 March 2025 Search strategy used: (“Platelet-Rich Plasma” OR “platelet rich plasma” OR “platelet-rich plasma” OR PRP) AND (tympanoplasty OR myringoplasty OR “fat graft myringoplasty” OR “tympanic membrane” OR “middle ear surgery” OR “sensorineural hearing loss” OR SSNHL OR ISSNHL OR “sudden hearing loss” OR intratympanic), with limits for publication dates (January 2015 to March 2025), English language, and human subjects. Records retrieved: 102

A3.2 Scopus

Date searched: 31 March 2025 Search strategy used: TITLE-ABS-KEY terms equivalent to PRP AND otologic surgery/hearing-loss terms (tympanoplasty, myringoplasty, fat graft myringoplasty, tympanic membrane, sensorineural hearing loss, SSNHL/ISSNHL, intratympanic), with limits for English language, publication years 2015-2025, and article document type. Records retrieved: 138

A3.3 Web of Science Core Collection

Date searched: 31 March 2025 Search strategy used: Topic search combining PRP terms with otologic/hearing-loss terms (tympanoplasty, myringoplasty, tympanic membrane, sensorineural hearing loss, SSNHL/ISSNHL, intratympanic), refined by English language and article type, restricted to 2015-2025. Records retrieved: 74

A3.4 EMBASE

Date searched: 31 March 2025 Search strategy used: Emtree/free-text equivalent terms for platelet-rich plasma combined with otologic surgery and hearing-loss terms, restricted to humans, English language, article type, and 2015-2025 publication window. Records retrieved: 86

A3.5 Cochrane Library

Date searched: 31 March 2025 Search strategy used: PRP terms combined with tympanoplasty/myringoplasty/tympanic membrane/hearing-loss/intratympanic terms within the 2015-2025 period. Records retrieved: 18

A4. Manual Searching

Reference lists of included studies and relevant reviews were manually screened for additional eligible studies not captured in primary database searches. Additional records identified by manual searching: 6

A5. Record Management

All records were imported into EndNote X9. Duplicates were removed using automated and manual verification before screening. Total records before duplicate removal: 424 Duplicate records removed: 64 Records after duplicate removal: 360 Full-text articles excluded due to paywall/non-retrievable access: 302 Full-text articles assessed for eligibility: 58 Full-text articles excluded after eligibility assessment: 52

Review/editorial format: 20

Insufficient or unclear outcome data: 18

Did not meet inclusion criteria: 14 Studies included in qualitative synthesis: 6

Appendix B. Inter-reviewer reliability and screening governance

Two reviewers independently screened studies at title/abstract and full-text levels. Disagreements were resolved through discussion; unresolved conflicts were adjudicated by a third reviewer.

**Table 4:** Inter-reviewer agreement (Cohen’s kappa) *Interpretation based on Landis and Koch categories.

Screening stage	Number screened (n)	Cohen’s kappa (κ)	Interpretation*
Title/Abstract	360	0.84	Almost perfect agreement
Full-text	58	0.79	Substantial agreement

B1. Screening Decision Rules

Records were excluded if they were non-original articles (e.g., reviews/editorials), non-human or in vitro studies, not focused on otologic PRP use, lacked relevant clinical outcomes, used ineligible designs, or had unretrievable full text after reasonable retrieval attempts.

Appendix C. Semi-quantitative synthesis framework

Because of heterogeneity in study design, PRP preparation, intervention delivery, and outcome reporting, pooled meta-analysis was not performed. A structured semi-quantitative narrative framework was used.

Studies were grouped by outcome domain:

Graft uptake/closure outcomes

Healing and postoperative complications/infection outcomes

Hearing outcomes (including SNHL-related outcomes)

For each domain, findings were coded by direction of effect:

PRP favored

No clear difference

Comparator favored

Statistical support was recorded as:

Significant difference reported

Non-significant difference reported

Not reported

Interpretation was design-aware (randomized evidence weighted more strongly than non-randomized/retrospective evidence) and integrated with risk-of-bias judgments.

Appendix D. Study-level statistical extraction

**Table 5:** Quantitative/statistical extraction from included studies “Not reported” indicates that the original study did not provide that statistic in a form extractable for this review’s comparative table.

Study	Design	Sample (PRP / comparator)	Clinical context	Primary endpoint(s)	PRP result	Comparator result	Absolute difference / effect estimate	p-value	95% CI
El-Anwar et al. [12]	Prospective clinical study	Not reported in extractable PRP/control format (single-arm clinical application)	Myringoplasty	Closure, complications	High closure; minimal complications	Not applicable	Not applicable	Not reported	Not reported
Fawzy et al. [13]	Prospective comparative study	Total n=60 (group-level split not consistently extractable in manuscript table)	Myringoplasty (CSOM)	Graft uptake	Higher uptake	Lower uptake	Direction favors PRP; numeric absolute difference not consistently reported in manuscript	<0.05	Not reported
Fouad et al. [14]	Retrospective comparative study	Total n=69 across PRP, HA, and control groups	Fat graft myringoplasty	Closure rate	85.7%	60% (control)	+25.7 percentage points (PRP vs control)	<0.05	Not reported
Mekhemar et al. [3]	Prospective clinical study	Total n=40 (group-level split not consistently extractable in manuscript table)	Myringoplasty	Healing, infection	Better healing; fewer infections	Less favorable healing; more infections	Direction favors PRP; numeric effect not reported	Not reported	Not reported
Vignesh et al. [9]	Single-blinded randomized controlled trial	25 / 25	Myringoplasty	Graft success	Higher success	Lower success	Direction favors PRP; numeric effect not reported in manuscript text/table	<0.05	Not reported
Shawky [15]	Prospective comparative clinical study	Total n=100 (PRP vs steroid comparator; subgroup sizes not reported in manuscript table)	Idiopathic sudden SNHL	Hearing improvement	Better/comparable hearing gain	Steroid group: moderate gain	Direction favors or is comparable for PRP; numeric effect not fully reported	Not reported	Not reported
